# Factors associated with the use of annual health checkups in Thailand: evidence from a national cross-sectional health and welfare survey

**DOI:** 10.3389/fpubh.2024.1390125

**Published:** 2024-05-10

**Authors:** Seung Chun Paek, Ning Jackie Zhang

**Affiliations:** ^1^Department of Society and Health, Faculty of Social Sciences and Humanities, Mahidol University, Salaya, Thailand; ^2^Health Solution Research Unit, Faculty of Social Sciences and Humanities, Mahidol University, Salaya, Thailand; ^3^College of Health Professions, Grand Valley State University, Grand Rapids, MI, United States

**Keywords:** annual health checkups, routine medical examinations, socioeconomic status, health risk behaviors, health status, Thailand

## Abstract

**Introduction:**

Thailand has provided free annual health checkups (AHC) since universal health coverage began in 2002. However, evidence regarding the equitable use of AHC is scarce. Thus, this study explored factors associated with the use of AHC in Thailand.

**Methods:**

A cross-sectional study was conducted using data from the national 2015 Health and Welfare Survey. Respondents aged 15 years or above (*n* = 57,343) were selected as the study sample. Descriptive statistical analysis and multivariable binary logistic regression were conducted to examine the association between the use of AHC and factors selected on the basis on Andersen’s Behavioral Model of Access to Medical Care.

**Results:**

Among the study sample, approximately 4.9% (*n* = 2,815) had used AHC during the past year. Regarding predisposing factors, the use of AHC was positively related to age (e.g., over 61 vs. 15–30: AOR = 2.90 [95% CI = 2.40–3.52], *p* < 0.001) and female sex (AOR = 1.23 [95% CI = 1.12–1.35] *p* < 0.001). For enabling factors, the AHC use was positively associated with income (e.g., Q4 vs. Q1: AOR = 1.98 [95% CI = 1.75–2.25], *p* < 0.001), education (e.g., high vs. low: AOR = 3.11 [95% CI = 2.75–3.51], *p* < 0.001), being married (e.g., vs. single: AOR = 1.27 [95% CI = 1.11–1.46], *p* < 0.001), and urban residency (AOR = 1.12 [95% CI = 1.04–1.22], *p* = 0.006). For need-for-care factors, the AHC use was positively related to chronic disease (AOR = 1.26 [95% CI = 1.15–1.38], *p* < 0.001), non-smoking (e.g., vs. daily: AOR = 1.18 [95% CI = 1.03–1.36], *p* = 0.015), non-drinking (e.g., vs. 3–4 times per week: AOR = 1.26 [95% CI = 1.04–1.53], *p* = 0.016), and vegetable consumption (e.g., over 5 ladles vs. below 1 ladle: AOR = 1.86 [95% CI = 1.50–2.31], *p* < 0.001).

**Conclusion:**

The results indicate that health awareness could play a significant role in the use of AHC. Individuals with high socioeconomic status (e.g., high-income people) and those with low-risk health behaviors (e.g., non-smokers) generally have a high interest in health and well-being. This may have been the primary reason for the high use of AHC among these groups. Thus, the government should continue to promote the public’s health awareness through various public campaigns and education programs to increase the use of AHC.

## 1 Introduction

The global disease burden has been steadily increasing along with the aging population and the corresponding rise of non-communicable diseases (NCDs) (1). According to the World Health Organization (2), in 2023, over 40 million people die every year due to NCDs, representing approximately 75% of all deaths globally. The main NCDs are cardiovascular disease, cancer, chronic respiratory disease, and diabetes, which together account for almost 80% of all global NCDs.

Consistent with the global trend, Thailand is facing an increasing aging population and NCDs. In 2022, people aged 65 years or over accounted for approximately 15% of the total population, the highest percentage in Southeast Asia (3). NCDs are the largest contributors to the increase of the burden of disease in Thailand, killing 400,000 people every year, which is equivalent to approximately 75% of all deaths in the nation. Cardiovascular disease, diabetes, cancer, and chronic obstructive pulmonary disease are four main NCDs in Thailand, accounting for over 90% of all NCDs (4).

Increasing attention has been paid to preventive care as a cost-effective intervention to decrease disease burden. Health checkups, which are a form of secondary preventive care designed to detect diseases early to stop or delay their progress, have become a key consideration among public health policymakers worldwide (5). Previous studies have indicated that using health checkups can increase the likelihood of positive health outcomes (6–8) and prevent healthcare expenditures for future diseases (7, 9–11).

Thailand achieved universal health coverage in 2002 and has since provided free annual health checkups (AHC) as a national strategy to promote the population’s health (12). However, evidence regarding the equitable use of AHC is scarce. To our knowledge, three studies have examined the use of health checkups and their influencing factors. Only one of these studies analyzed the use of AHC for older adults (13), while the other two analyzed the use of cervical cancer screening tests for middle-aged women (14, 15).

The overall pattern indicates that use of health checkups tends to be high among high socioeconomic groups. People with high socioeconomic status (e.g., high-income people), low-risk health behaviors (e.g., non-smokers), and low health status (e.g., chronic patients) used AHC, which is the primary variable of interest in this study, more than their counterparts (13).

These previous studies offer a comprehensive understanding of the use of health checkups and their associated factors. However, as mentioned previously, only one study examined the use of AHC. Moreover, all three previous studies mentioned above were performed using limited samples from particular groups (i.e., older adults and middle-aged women) in specific regions. Evidence from nationally representative samples is required to verify the generalizability and reliability of previous findings for future policy developments to address socioeconomic inequalities in the use of AHC. Therefore, the present study used data from the national 2015 Health and Welfare Survey (HWS) to investigate the use of AHC and its associated factors in Thailand.

## 2 Literature review and research assumptions

Factors associated with the use of health checkups have been extensively studied in other countries. These can be divided into four groups: demographic characteristics, socioeconomic status, health risk behaviors, and health status. In line with this study’s objective, we conducted a literature review focusing on previous studies that were performed using (1) general health checkups (e.g., not disease-specific checkups such as cervical cancer screening tests), (2) the general population (e.g., not age- or gender-specific populations such as older adults and women), and (3) large-scale data (e.g., nationwide survey data).

Age and sex were frequently examined as demographic factors in previous studies. These studies commonly found that older adults and women tended to use health checkups more than younger adults and men, respectively (5, 16–20). Furthermore, the use of health checkups generally increased along with age, and this increase was significantly high for people older than 40–50 years.

Regarding socioeconomic status, the use of health checkups was high among high socioeconomic groups (5, 16–21). Although results varied across previous studies, income and education were consistently cited as positive determinants of the use of health checkups. In addition, people who were married (5, 18, 19, 21), employed (5, 16, 20, 21), insured (18), and urban residents (21) used health checkups more than their counterparts. These socioeconomic inequalities were also found in other studies examining disease-specific checkups for age or gender-specific groups such as middle-aged women and older adults (22–25).

Low-risk health behaviors were positively associated with the use of health checkups (5, 18–21). Previous studies defining smoking, drinking, dietary behaviors, and physical activity as health risk behaviors have shown that smoking and drinking were negatively related to the use of health checkups. Meanwhile, healthy dietary behaviors (e.g., high daily consumption of vegetables) and routine exercise were positively related to the use of health checkups.

Lastly, previous studies examining objective (e.g., chronic diseases) and subjective health status (e.g., self-rated health) indicated that low health status was related to the use of health checkups (5, 17–19). For instance, Culica et al. (18) showed that people with cardiovascular disease, diabetes, or hypertension were more inclined to use health checkups than those without these ailments. Moreover, those reporting “poor” or “fair” in the self-rated health assessment were more inclined to use health checkups than those reporting “good” or “excellent.” The positive relationship between low health status and high health checkup use was consistently found for objective health status but only occasionally for subjective health status.

These previous results can be understood based on the health belief model. According to this model, individuals will take action to improve their health if they perceive that they are susceptible to a disease (perceived susceptibility), the disease will lead to serious consequences (perceived severity), taking action will decrease the susceptibility or severity (perceived benefit), and there will not be obstacles to taking action (perceived barriers) (26, 27).

On the one hand, people with high socioeconomic status (e.g., high income) and those with low-risk health behaviors (e.g., routine exercise) who generally have a high interest in health and well-being probably have high perceived benefits. This may be the primary reason for the high use of health checkups among this group. On the other hand, people with low health status (e.g., chronic patients) who generally have concerns about their health problems probably have high perceived susceptibility and severity. This may increase the use of health checkups for this group of people.

In addition, people with health insurance and those who live in urban regions generally have low financial burdens of using healthcare and high physical accessibility to healthcare providers, respectively. Such low perceived barriers may increase their use of health checkups. Based on the literature review, this study attempted to establish and test the following four assumptions.

*Assumption 1*. Individuals with high socioeconomic status who generally have a high interest in health and well-being will likely use AHC. High-income, high-educated, married, and employed people were considered high socioeconomic groups in this study.

*Assumption 2*. Individuals with low-risk health behaviors who generally have a high interest in health and well-being will likely use AHC. Non-smokers, non-drinkers, and individuals with healthy dietary behaviors (i.e., low sugary drink consumption and high vegetable consumption) were considered low-risk health behavior groups in this study.

*Assumption 3*. Individuals with low health status who are generally concerned about their health problems will likely use AHC. Older adults, women, chronic patients, and people with low self-rated health status (i.e., low self-rated health and high self-rated depression) were considered low health status groups in this study.

*Assumption 4*. Individuals in urban areas with high healthcare provider density and affordable transportation options (28, 29) will likely use AHC.

## 3 Methods

### 3.1 Data and sample

This study used a cross-sectional design with data from the 2015 HWS, which is a nationwide biannual survey performed by the National Statistical Office of Thailand. The HWS contains comprehensive sets of questions categorized into four main modules, including (1) demographic and socioeconomic parameters, (2) illness and use of healthcare services, (3) health risk factors and behaviors, and (4) housing characteristics.

The goal of the HWS is to produce statistically reliable estimates for each indicator represented at the national level. To accomplish this goal, the survey uses a stratified two-stage sampling approach. Specifically, the scope of the survey covers households in all municipal (i.e., urban) and non-municipal (i.e., rural) areas in all 77 provinces of Thailand. The first stratum is all 77 provinces and the second stratum in each province has two sub-strata: municipal and non-municipal areas.

The first stage is to randomly select enumeration areas from all municipal and non-municipal areas based on proportional probability to the size of the population. The second stage is to randomly select 12–16 households from municipal enumeration areas and 8–12 households from non-municipal enumeration areas. Thus, the survey represents a national cross-section of all 77 provinces of Thailand, with approximately equal-sized samples from each province (30).

The National Statistical Office conducted the HWS in 2021 during the COVID-19 pandemic but did not release it. Furthermore, the 2017 and 2019 HWSs, which provide the most recent data, do not include information regarding health risk behaviors or health status. Thus, the 2015 HWS used in this study is the most recent survey that includes all variables of interest, when this study was performed. In the 2015 HWS, respondents aged 15 years or above were asked whether they had ever used AHC during the past year; accordingly, a total of 57,343 individuals aged 15 years or above were selected as the study sample for analysis.

### 3.2 Variables and measurement

The dependent variable in this study was the use of AHC and was measured as a binary variable (yes and no). In the 2015 HWS, respondents aged 15 years or above were asked whether they had ever used AHC during the past year. Those who answered “yes” and “no” to the question were classified into the “yes” and “no” groups in the dependent variable, respectively.

In addition, this study selected the independent variables based on Andersen’s access to medical care model. According to this model, the factors affecting healthcare access are classified into predisposing, enabling, and need-for-care factors. Predisposing factors are the demographic and sociocultural characteristics of individuals before the onset of illness. Enabling factors are individual- and community-level resources that facilitate access to healthcare. Need-for-care factors are perceived (subjective) and evaluated (objective) health problems or illness levels that generate the need for healthcare (31).

In this study, two demographic variables (age and sex) and five socioeconomic variables (income, education, employment, marital status, and place of residence) were selected as predisposing and enabling factors, respectively. Three health statuses (chronic disease, self-rated health, and self-rated depression) and four health risk behaviors (smoking, drinking, vegetable consumption, and sugary drink consumption) were selected as need-for-care factors (19, 31).

Age was measured as an ordinal variable with five categories (15–30, 31–40, 41–50, 51–60, and 61 years or above). Sex was used as a binary variable (male and female). Income was measured in four quartiles (Q1 to Q4) based on household equivalent income (32). Marital status was used as a nominal variable with three categories (single, married, and divorced). The “divorced” category included people who were divorced, separated, or widowed. Education was measured as an ordinal variable with three categories (low, middle, and high). The “low,” “middle,” and “high” categories meant primary school or below, secondary school, and college or above, respectively. Employment (yes and no) and place of residence (urban and rural) were measured as binary variables.

Smoking was an ordinal variable with three categories (no, occasional, daily), as was drinking (no, 1–7 times per month, and 3–4 times per week). Sugary drink consumption was measured as an ordinal variable with four categories (no, less than 1 bottle, 1–2 bottles, and more than 2 bottles per day). Vegetable consumption was measured as an ordinal variable with five groups (less than 1 ladle, 1 ladle, 2–3 ladles, 4–5 ladles, and more than 5 ladles per day).

Chronic disease was measured as a binary variable (yes and no). Self-rated health was an ordinal variable with three categories (worse, same, and better), as was self-rated depression (not at all, slightly, and moderately or above). Regarding self-rated health, respondents were asked to rate their overall health compared to others of their age and sex on a five-point scale: much worse, slightly worse, same, slightly better, and much better. However, due to low frequencies of the “much worse” and “much better” categories, we combined them with the “slightly worse” and “slightly better” categories and labeled these combined categories “worse” and “better,” respectively. In terms of self-rated depression, respondents were asked to rate their depression levels on a five-point scale: not at all, slightly, moderately, very, and extremely. Due to the same issue that we encountered for overall health, we combined the “moderately,” “very,” and “extremely” responses and labeled this combined category “moderately or above.”

In summary, age (five categories), income (four categories), education (three categories), smoking (three categories), drinking (three categories), sugary drink consumption (four categories), vegetable consumption (five categories), self-rated health (three categories), and self-rated depression (three categories) were used as ordinal scale variables. Marital status (three categories) was used as a nominal scale variable. Sex, employment, place of residence, and chronic disease were used as binary variables for analysis.

### 3.3 Statistical analysis

Descriptive analysis was conducted to summarize the study sample and variables. The bivariate relationship between the use of AHC and each independent variable was investigated by a chi-squared test. In addition, since the use of AHC was a binary variable, a multivariable binary logistic regression (BLR) was used to investigate multivariate relationships between the outcome and independent variables. The performance of the BLR model was evaluated using Hosmer and Lemeshow’s goodness-of-fit test (33).

In addition, following the guidelines suggested by Alkan et al. (34), this study tested four essential assumptions of the BLR model: the independence of errors, the lack of multicollinearity, the lack of outliers, and linearity. First, the independence of errors was tested using the ratio of the deviance and Pearson goodness-of-fit chi-square values to the degrees of freedom (DF) of the model. The ratio of the deviance value to the DF was 0.43 (deviance value = 14,476.89 with 33,333 DF) and the ratio of the Pearson goodness-of-fit chi-square value to the DF was 0.98 (Pearson chi-square value = 32,651.34 with 33,333 DF). Since both ratios were less than 1, the independence of errors was not an issue (34).

Second, multicollinearity among the independent variables was assessed using the variance inflation factor and cross-comparison between the crude and adjusted odds ratios. The score of the variance inflation factor ranged from 1.06 to 3.77, and there were no large directional changes between the crude and adjusted odds ratios, suggesting that multicollinearity was not a problem (34, 35).

Third, outliers were explored using Pearson and deviance residuals. If a case has an absolute value greater than 3, the case can be considered an outlier (34). In this study, the BLR model included a total of 57,343 cases. Among them, 5 cases had an absolute value greater than 3 in the deviance residual and 287 cases had that in the Pearson residual. However, these outlier cases were included in the BLR analysis.

Last, the assumption of linearity was not tested since all independent variables used in the BLR model were categorical (34). Statistical significance was fixed at a *p*-value of 0.05. For the BLR analysis, the odds ratio and a 95% confidence interval (CI) were used to determine the directional relationship and its statistical significance, respectively. IBM SPSS Statistics 20 software was used for all statistical analyses.

## 4 Results

### 4.1 Descriptive statistical analysis

The results of the descriptive analysis are presented in Table 1. Among the study sample (i.e., individuals aged 15 years or above, *n* = 57,343), approximately 4.9% (*n* = 2,815) used AHC during the past year. Moreover, as expected, the results of the bivariate association from the chi-squared test indicated that the rate of AHC use was higher in people who had high socioeconomic status, low-risk health behaviors, low health status, and lived in urban areas than their counterparts.

**Table 1:** Results of descriptive statistical analysis.

			Use of Annual Health Checkups				*X*^2^ test *p*-value
	Overall		Yes (*N* = 2,815; 4.9%)		No (*N* = 54,528; 95.1%)		
Variables	*n*	%	*n*	%	*n*	%	
**Predisposing factors**							
Age group							<0.001^*^
15–30	7,703	13.4	211	2.7	7,492	97.3	
31–40	8,945	15.6	352	3.9	8,593	96.1	
41–50	12,677	22.1	596	4.7	12,081	95.3	
51–60	13,078	22.8	800	6.1	12,278	93.9	
Over 61	14,940	26.1	856	5.7	14,084	94.3	
Sex							0.010^*^
Male	23,068	40.2	1,067	4.6	22,001	95.4	
Female	34,275	59.8	1,748	5.1	32,527	94.9	
**Enabling factors**							
Income quartile							<0.001^*^
Q1	16,198	28.3	554	3.4	15,644	96.6	
Q2	13,782	24.0	470	3.4	13,312	96.6	
Q3	13,656	23.8	544	4.0	13,112	96.0	
Q4	13,707	23.9	1,247	9.1	12,460	90.9	
Education							<0.001^*^
Low	35,515	61.9	1,382	3.9	34,133	96.1	
Middle	16,114	28.1	703	4.4	15,411	95.6	
High	5,714	10.0	730	12.8	4,984	87.2	
Marital status							<0.001^*^
Single	8,175	14.3	309	3.8	7,866	96.2	
Married	38,835	67.7	2,043	5.3	36,792	94.7	
Divorced	10,333	18.0	463	4.5	9,870	95.5	
Employment							<0.001^*^
Yes	44,040	76.8	2,049	4.7	41,991	95.4	
No	13,303	23.2	766	5.8	12,537	94.2	
Place of residence							<0.001^*^
Urban	31,511	55.0	1,785	5.7	29,726	94.3	
Rural	25,832	45.1	1,030	4.0	24,802	96.0	
**Need-for-care factors**							
Smoking							<0.001^*^
No	47,096	82.1	2,475	5.3	44,621	94.7	
Occasional	1,219	2.1	42	3.5	1,177	96.6	
Daily	9,028	15.7	298	3.3	8,730	96.7	
Drinking							<0.001^*^
No	39,450	68.8	1,965	5.0	37,485	95.0	
1–7 times per month	13,854	24.2	716	5.2	13,138	94.8	
3–4 times per week	4,039	7.0	134	3.3	3,905	96.7	
Sugary drink consumption							0.603
No	20,027	34.9	961	4.8	19,066	95.2	
Less than 1 bottle	16,465	28.7	766	4.7	15,699	95.4	
1–2 bottles	18,386	32.1	954	5.2	17,432	94.8	
More than 2 bottles	2,465	4.3	134	5.4	2,331	94.6	
Vegetable consumption							<0.001^*^
Less than 1 ladle	3,270	5.7	115	3.5	3,155	96.5	
1 ladle	11,658	20.3	439	3.8	11,219	96.2	
2–3 ladles	25,838	45.1	1,286	5.0	24,552	95.0	
4–5 ladles	10,973	19.1	569	5.2	10,404	94.8	
More than 5 ladles	5,604	9.8	406	7.2	5,198	92.8	
Chronic disease							<0.001^*^
Yes	16,787	29.3	1,028	6.1	15,759	93.9	
No	40,556	70.7	1,787	4.4	38,769	95.6	
Self-rated health							0.367
Worse	5,038	8.8	264	5.2	4,774	94.8	
Same	24,476	42.7	1,173	4.8	23,303	95.2	
Better	27,829	48.5	1,378	5.0	26,451	95.1	
Self-rated depression							0.645
Not at all	47,668	83.1	2,355	4.9	45,313	95.1	
Slightly	8,137	14.2	391	4.8	7,746	95.2	
Moderately or above	1,538	2.7	69	4.5	1,469	95.5	

^*^statistically significant at 0.05.

Specifically, regarding socioeconomic status, as expected from Assumption 1, the rate of AHC use was higher among high-income (e.g., Q4: 9.1% vs. Q1: 3.4%), high-educated (e.g., high: 12.8% vs. low: 3.9%), and married people (e.g., married: 5.3% vs. single: 3.8%). However, contrary to Assumption 1, employment was negatively related to the use of AHC. That is, unemployed people (5.8%) had a higher rate of AHC use than employed people (4.7%).

In terms of health risk behaviors, consistent with Assumption 2, the rate of AHC use was higher among individuals who did not smoke (e.g., no: 5.3% vs. daily: 3.3%), did not drink (e.g., no: 5.0% vs. 3–4 times per week: 3.3%), and had a high vegetable consumption (e.g., more than 5 ladles: 7.2% vs. less than 1 ladle: 3.5%). However, sugary drink consumption was not significantly related to the use of AHC.

In the case of health status, as expected per Assumption 3, the rate of AHC use was higher among older people (e.g., over 61: 5.7% vs. 15–30: 2.7%), females (5.1% vs. male: 4.6%), and those with a chronic disease (6.1% vs. those without a chronic disease: 4.4%). However, subjective health status (i.e., self-rated health and depression) was not significantly related to the use of AHC. Lastly, consistent with Assumption 4, urban residents (5.7%) had a higher rate of AHC use than rural residents (4.0%).

### 4.2 Binary logistic regression analysis

Table 2 reveals the results of the BLR analysis. The *p*-value of Hosmer and Lemeshow’s goodness-of-fit test was 0.16, indicating that the BLR models did not show a lack of fit with the data (33). Similar to the descriptive analysis, the BLR model indicated that high socioeconomic status, low-risk health behaviors, low health status, and urban residency were positively related to the use of AHC.

**Table 2:** Results of binary logistic regression analysis.

Variables	COR	95% CI	*p*-value	AOR	95% CI	*p*-value	VIF
**Predisposing factors**							
Age group							
31–40	1.45	(1.22, 1.73)	<0.001^*^	1.33	(1.11, 1.60)	0.002^*^	2.11
41–50	1.75	(1.49, 2.06)	<0.001^*^	1.95	(1.63, 2.32)	<0.001^*^	2.75
51–60	2.31	(1.98, 2.70)	<0.001^*^	2.69	(2.25, 3.21)	<0.001^*^	3.11
Over 61	2.16	(1.85, 2.52)	<0.001^*^	2.90	(2.40, 3.52)	<0.001^*^	3.53
15–30	1.00			1.00			
Sex							
Female	1.11	(1.03, 1.20)	0.010^*^	1.23	(1.12, 1.35)	<0.001^*^	1.49
Male	1.00			1.00			
**Enabling factors**							
Income quartile							
Q2	1.00	(0.88, 1.13)	0.962	1.06	(0.93, 1.20)	0.397	1.48
Q3	1.17	(1.04, 1.32)	0.010^*^	1.20	(1.06, 1.36)	0.005^*^	1.56
Q4	2.83	(2.55, 3.13)	<0.001^*^	1.98	(1.75, 2.25)	<0.001^*^	1.90
Q1	1.00			1.00			
Education							
Middle	1.13	(1.03, 1.24)	0.012^*^	1.44	(1.29, 1.61)	<0.001^*^	1.52
High	3.62	(3.29, 3.98)	<0.001^*^	3.11	(2.75, 3.51)	<0.001^*^	1.46
Low	1.00			1.00			
Marital status							
Married	1.41	(1.25, 1.60)	<0.001^*^	1.27	(1.11, 1.46)	<0.001^*^	2.41
Divorced	1.19	(1.03, 1.38)	0.018^*^	1.02	(0.86, 1.20)	0.840	2.54
Single	1.00			1.00			
Employment							
No	1.25	(1.15, 1.36)	<0.001^*^	1.30	(1.18, 1.43)	<0.001^*^	1.27
Yes	1.00			1.00			
Place of residence							
Urban	1.45	(1.34, 1.56)	<0.001^*^	1.12	(1.04, 1.22)	0.006^*^	1.06
Rural	1.00			1.00			
**Need-for-care factors**							
Smoking							
No	1.63	(1.44, 1.84)	<0.001^*^	1.18	(1.03, 1.36)	0.015^*^	1.47
Occasional	1.05	(0.75, 1.45)	0.791	0.90	(0.65, 1.26)	0.546	1.12
Daily	1.00			1.00			
Drinking							
No	1.53	(1.28, 1.83)	<0.001^*^	1.26	(1.04, 1.53)	0.016^*^	2.39
1–7 times per month	1.59	(1.32, 1.92)	<0.001^*^	1.60	(1.32, 1.94)	<0.001^*^	2.07
3–4 times per week	1.00			1.00			
Sugary drink consumption							
No	0.88	(0.73, 1.06)	0.165	0.93	(0.77, 1.13)	0.470	3.51
Less than 1 bottle	0.85	(0.70, 1.03)	0.088	0.95	(0.78, 1.16)	0.601	3.40
1–2 bottles	0.95	(0.79, 1.15)	0.604	1.01	(0.84, 1.23)	0.902	3.77
More than 2 bottles	1.00			1.00			
Vegetable consumption							
More than 5 ladles	2.14	(1.73, 2.65)	<0.001^*^	1.86	(1.50, 2.31)	<0.001^*^	1.50
4–5 ladles	1.50	(1.22, 1.84)	<0.001^*^	1.34	(1.09, 1.65)	0.006^*^	2.14
2–3 ladles	1.44	(1.18, 1.75)	<0.001^*^	1.34	(1.10, 1.63)	0.004^*^	2.96
1 ladle	1.07	(0.87, 1.32)	0.506	1.08	(0.88, 1.34)	0.462	2.19
Less than 1 ladle	1.00			1.00			
Chronic disease							
Yes	1.42	(1.31, 1.53)	<0.001^*^	1.26	(1.15, 1.38)	<0.001^*^	1.93
No	1.00			1.00			
Self-rated health							
Slightly or much worse	1.06	(0.93, 1.22)	0.387	1.02	(0.87, 1.20)	0.803	1.46
Same	0.97	(0.89, 1.05)	0.399	0.94	(0.86, 1.03)	0.158	1.28
Slightly or much better	1.00			1.00			
Self-rated depression							
Moderately or above	0.97	(0.87, 1.08)	0.602	0.99	(0.88, 1.11)	0.807	1.12
Slightly	0.90	(0.71, 1.16)	0.418	0.93	(0.72, 1.20)	0.586	1.09
Not at all	1.00			1.00			
Hosmer-Lemeshow GOF test							
Chi-squared score (DF)				11.86 (8)			
*p*-value				0.158			

^*^statistically significant at 0.05; COR and AOR, crude and adjusted odds ratio; CI, confidence interval; GOF, goodness-of-fit; VIF, variance inflation factor.

Regarding socioeconomic status, consistent with Assumption 1, high-income, high-educated, and married people used AHC more than their counterparts. In terms of income, the odds of using AHC were 1.98 (95% CI = 1.75–2.25, *p*-value<0.001) and 1.20 (95% CI = 1.06–1.36, *p*-value = 0.005) times higher in people in Q4 and Q3 than in those in Q1. However, the odds of using AHC were not significantly different between people in Q2 and Q1. Regarding education, the odds of using AHC were 3.11 (95% CI = 2.75–3.51, *p*-value<0.001) and 1.44 (95% CI = 1.29–1.61, p-value<0.001) times higher in High- and middle-educated people than in low-educated people. For marital status, the odds of using AHC were 1.27 times higher in married people than in single people, with a 95% CI of 1.11 to 1.46 and a *p*-value of <0.001. However, the odds of using AHC were not different between single and divorced people. Regarding employment, contrary to Assumption 1, the odds of using AHC were 1.30 times higher in unemployed individuals than in employed individuals, with a 95% CI of 1.18 to 1.43 and a *p*-value of <0.001.

In terms of health risk behaviors, as expected from Assumption 2, individuals who did not smoke, did not drink, and had a high vegetable consumption used AHC more than their counterparts. Specifically, the odds of using AHC were 1.18 times higher in non-smokers than in daily smokers, with a 95% CI of CI = 1.03 to 1.36 and a *p*-value of 0.015. However, daily and occasional smokers did not significantly differ in their use of AHC. Regarding drinking, the odds of using AHC were 1.26 (95% CI = 1.04–1.53, *p*-value = 0.016) and 1.60 (95% CI = 1.32–1.94, *p*-value<0.001) times higher in people who did not drink and who drank 1–7 times per month than in those who drank 3–4 times per week. In terms of vegetable consumption, the odds of using AHC were 1.86 (95% CI = 1.50–2.31, *p*-value<0.001), 1.34 (95% CI = 1.09–1.65, *p*-value = 0.006), and 1.34 (95% CI = 1.10–1.63, *p*-value = 0.004) times higher in people consuming more than 5 ladles, 4–5 ladles, and 2–3 ladles than in those consuming less than 1 ladle. However, people consuming 1 ladle and those less than 1 ladle were not significantly different in their use of AHC. Sugary drink consumption was not significantly associated with the use of AHC.

Regarding health status, as expected based on Assumption 3, people with low health status (i.e., older adults, women, and chronic patients) tended to use AHC more than their counterparts. Specifically, the odds of using AHC were 1.33 (95% CI = 1.11–1.60, *p*-value = 0.002), 1.95 (95% CI = 1.63–2.32, *p*-value<0.001), 2.69 (95% CI = 2.25–3.21, *p*-value<0.001), and 2.90 (95% CI = 2.40–3.52, *p*-value<0.001) times higher in people aged 31–40, 41–50, 51–60, and 61 years or above than in those aged 15–30 years. The odds of using AHC were 1.23 (95% CI = 1.12–1.35, p-value<0.001) and 1.26 (95% CI = 1.15–1.38, *p*-value<0.001) times higher in women and chronic patients than men and non-chronic patients, respectively. Self-rated health and depression were not significantly related to the use of AHC. Lastly, regarding place of residence, as expected per Assumption 4, the odds of using AHC were 1.22 times higher in urban residents than in rural residents, with a 95% CI of 1.12 to 1.35 and a *p*-value of 0.006.

## 5 Discussion

The present study explored the use of AHC and its associated factors among individuals aged 15 years or above in Thailand. Descriptive analysis and multivariable BLR were performed using data from the 2015 HWS, which is the most recent survey that includes all variables of interest in this study.

The results reveal that among the study sample (*n* = 57,343), approximately 4.9% (*n* = 2,815) had used AHC during the past year. Moreover, as expected given this study’s four evidence-based assumptions established through a literature review, AHC use was significantly high among people with high socioeconomic status (i.e., high-income, high-educated, and married individuals), low-risk health behavior (i.e., non-smokers, non-drinkers, and those with high vegetable consumption), and low health status (i.e., older adults, women, and chronic patients), as well as urban residents. However, contrary to this study’s expectation, employment was reversely related to the AHC use. Moreover, sugary drink consumption and self-rated health and depression were not significantly related to the AHC use.

The results indicate that health awareness could play a significant role in the use of AHC. As previously explained in the establishment of the assumptions in this study, individuals with high socioeconomic status and low-risk health behaviors generally have a high interest in health and well-being, while those with low health status generally have concerns about their health. This may have been the primary reason for the high use of AHC among these groups. Therefore, the government should continue public campaigns and education to promote the public’s health awareness, thereby increasing the use of AHC. This study also found that low-risk health behaviors (primary prevention) are positively related to the use of AHC (secondary prevention). This indicates that strengthening current healthy lifestyle campaigns, such as anti-smoking and low-sodium campaigns, is expected to increase the use of AHC.

In addition, this study found a low use of AHC among rural residents, as expected based on Assumption 4. Geographical barriers such as long distances to healthcare providers and a lack of affordable transportation options are well-known factors that hamper healthcare access in Thailand (28, 29). Thus, the government’s investment in current insufficient healthcare resources and infrastructure in rural and remote areas should be expanded to mitigate the urban–rural gap in the use of AHC.

Contrary to Assumption 1, employment was negatively related to the use of AHC. That is, employed people used AHC less than unemployed people. This low accessibility of the employed group is consistent with previous studies examining healthcare access in Thailand (12, 36). These studies found that employed people were less likely than unemployed people to use institutional healthcare services (i.e., services from healthcare providers) due to time constraints during the day and the limited service hours of healthcare providers (from 9:00 a.m. to 5:00 p.m.). Employed people were also more likely to use informal care services or forego healthcare services, which should be considered by the government.

In addition, sugary drink consumption was not significantly associated with the use of AHC. This is probably due to the measurement imprecision of this variable, as indicated in a previous study (37). That is, the questionnaire related to sugary drink consumption in the 2015 HWS did not distinguish between sugar-sweetened and artificially sweetened beverages, and this measurement ambiguity may have resulted in a non-significant relationship. Further, to our knowledge, no studies have analyzed sugary drink consumption and the use of health checkups. Thus, empirical studies addressing the measurement issue are needed to examine the reliability and validity of this result.

Furthermore, this study found that chronic disease was related to the use of AHC, while self-rated health and depression were not. This result is consistent with previous studies (5, 17) and indicates that AHC use could be influenced by objective health status rather than subjective health status in Thailand. Of note, a single-item scale was used in this study to measure the subjective health status, and future studies should consider valid multiple-item measures, such as the Center for Epidemiological Studies Depression Scale, to estimate the precise impact of subjective health status on the use of AHC.

This study has limitations that should be addressed in future studies. First, the 2015 HWS used in this study is relatively outdated; accordingly, this study’s results may not reflect current patterns of the use of AHC and its associated factors. Thus, the reliability and validity of this study’s results should be evaluated using a more recent HWS. The National Statistical Office of Thailand must consider including information regarding health status and health risk behaviors in future HWS. Nevertheless, considering regular national survey data are stable and consistent across years in general, this study can be significant as the first study to show trends in the use of AHC for the entire Thai population while considering diverse socio-demographic characteristics, health risk behaviors, and health status.

Second, this study did not consider a weighted analysis since the 2015 HWS did not provide sample weights. Although the survey represents a national cross-section of all 77 provinces of Thailand with almost equal-sized samples from each province, a weighted analysis is necessary to reduce the bias and ensure the representativeness of estimates. This issue also must be considered by the National Statistical Office of Thailand.

Third, this study’s evidence-based assumptions were established indirectly using the view of the health belief model (i.e., people’s health awareness, such as perceived benefits of health-promoting behaviors, increases the use of AHC) and, accordingly, were tested implicitly. Thus, this study still provides a limited understanding of whether, for instance, the low AHC use among low-income people is due to the perceived benefits of using AHC, the perceived barriers to using AHC, or both. An empirical study examining associations between socio-demographic factors and the theoretical components of the health belief model could address this methodological limitation.

Fourth, since the cross-sectional design used in this study includes the issue of simultaneity (19), causal relationships for certain variables may have been reversed. For instance, people who used AHC may have exhibited low-risk health behaviors as a result of their AHC use. That is, people may have changed their unhealthy lifestyle to a healthy lifestyle (e.g., quitting smoking and improving eating habits) after their AHC use. Due to this simultaneity issue, this study may have underestimated or overestimated the impact of health risk behaviors on AHC use. Thus, this study’s results should be interpreted with caution.

Lastly, a longitudinal study should be performed to evaluate whether and how the cross-sectional patterns in AHC use found in this study change over time. This would present a methodological alternative to addressing the simultaneity issue mentioned in the third limitation above.

## 6 Conclusion

This study explored factors associated with the use of AHC among individuals aged 15 years or above in Thailand. The results reveal that approximately 4.9% (*n* = 2,815) of the study sample (*n* = 57,343) used AHC during the last year. As expected given this study’s four evidence-based assumptions, AHC use was significantly high among high socioeconomic individuals (i.e., high-income, high-educated, and married individuals), people with low-risk health behaviors (i.e., non-smokers, non-drinkers, and those with high vegetable consumption), people with low health status (i.e., older adults, women, and chronic patients), and urban groups. Contrary to this study’s expectation, employment was reversely related to the use of AHC. Meanwhile, sugary drink consumption and self-rated health and depression were not significantly related to the use of AHC. Health awareness may have played a significant role in the use of AHC.

The results indicate that health awareness could play a significant role in the use of AHC. Individuals with high socioeconomic status and low-risk health behaviors generally have a high interest in health and well-being, while those with low health status generally have concerns about their health. This may have been the primary reason for the high use of AHC among these groups. Thus, the government should continue to promote the public’s health awareness through various public campaigns and education programs to increase the use of AHC.

## Data availability statement

The data analyzed in this study is subject to the following licenses/restrictions: The dataset that supports the findings of this study is available from the National Statistical Office of Thailand. However, restrictions apply regarding the availability of the dataset, which was used under license for the present study and, thus, is not publicly available. However, the dataset is available from the authors upon reasonable request and with the permission of the National Statistical Office of Thailand. Requests to access these datasets should be directed to SCP, seungchun.pak@mahidol.ac.th.

## Ethics statement

This study was granted “Exemptions from IRB review” by the Office of the Committee for Research Ethics of the Faculty of Social Sciences and Humanities, Mahidol University (Certificate of Exemption No.: 2021/008.1604).

## Author contributions

SCP: Conceptualization, Data curation, Formal analysis, Investigation, Methodology, Writing – original draft, Writing – review & editing. NZ: Conceptualization, Investigation, Methodology, Writing – review & editing.
